# Between empowerment, patronization, and surveillance. A semi-structured interview study with persons with dementia and family caregivers on the empowering opportunities and perils of intelligent assistive technologies

**DOI:** 10.1186/s12910-025-01203-7

**Published:** 2025-04-05

**Authors:** Clara Löbe, Niklas Petersen

**Affiliations:** 1 University Medical Center Göttingen, Göttingen, 37099 Germany; 2 Department of Medical Ethics and History of Medicine, Humboldtallee 36, Göttingen, 37073 Germany

**Keywords:** Intelligent Assistive Technology, Dementia, Empowerment, User-Centered Design, Health Technology, Privacy, GPS-Tracking, Technology Adoption

## Abstract

**Background:**

Intelligent assistive technology (IAT) can contribute to the empowerment of persons with dementia by increasing independence, strengthening social participation, and improving quality of life. IAT could, however, also create new dependencies, reinforce power asymmetries, perpetuate stigmatization, and invade the privacy of persons living with dementia. To fulfill the empowering promise of new technologies and design a user-friendly IAT, users'perspectives, needs, capabilities and interests should be incorporated into IAT development and implementation from an early stage. Yet, the development and ethical assessment of IAT still tends to neglect the perspectives of potential user groups. This study explores how persons with dementia and their caregivers assess the empowering potential, opportunities, and risks of IAT.

**Methods:**

We conducted a qualitative content analysis of 27 semi-structured interviews with persons with dementia (12) and their caregivers (15). Three technologies (GPS bracelet, dressing technology, and emotion recognition technology) were presented in the interviews using fictional case vignettes.

**Results:**

Persons with dementia and their caregivers generally believe that IAT can potentially empower persons with dementia by improving their independence in performing daily tasks, supporting their independent mobility, increasing their physical and emotional sense of safety, and improving their social participation. The risks they identify include violations of privacy, patronization through technology, lack of user specificity, and insufficient everyday usability. Technologies are viewed very differently depending on the context, purpose of use, and user group.

**Conclusion:**

IATs seem to have the potential to empower persons with dementia, but risks and benefits are perceived differently by the interviewees. The technology’s usefulness depends on adapting to users' needs, capabilities, and interests. Future studies using a participatory approach that includes user preferences from the outset could lead to more user-centered technologies that promote the empowerment of persons with dementia.

**Supplementary Information:**

The online version contains supplementary material available at 10.1186/s12910-025-01203-7.

## Background

Intelligent assistive technology (IAT), such as GPS devices, smart home systems, and memory aids are promoted as being able to empower persons with dementia, increase their autonomy, improve independent mobility and quality of life, and reduce caregiver burden [21], p.7,[26, 39, 40, 48, 52]. Some technology assessment studies have examined the attitudes of persons with dementia and caregivers toward IAT [2, 6, 25, 30, 35, 36, 40, 47]. Devices for emergency assistance, navigation, monitoring, reminders, and communication are seen as beneficial due to their positive impact on independence, safety, communication, and cognition [2, 25, 35, 40, 47]. They also help reduce worry and anxiety for both persons with dementia and their caregivers, who express a desire for the technology to ease their workload and be adaptable to users' needs [6, 30]. Despite the hoped-for positive effects of IAT on the daily lives of persons with dementia and the work of caregivers, IAT is also associated with ethical, social, and legal challenges [42, 53]. For example, some critics have asked whether IAT might limit the freedom and decision-making of persons with dementia, violate their privacy through constant surveillance, increase stigmatization, or create new dependencies [23, 42, 49]. Others have suggested that technologies that aim to restore functionality may reproduce a discriminating conception of normalcy, thus reinforcing power asymmetries and problematizing their impact [45].

As dementia care increasingly focuses on person-centered approaches and patient empowerment [18], interventions and strategies aimed at empowering individuals with dementia and helping them maintain meaningful lives for longer periods have gained attention [51]. The term "empowerment" encompasses approaches in psychosocial practice that aim to support people in achieving change in their lives, making self-determined decisions, strengthening participation, and increasing independence in everyday life [3, 11, 14]. The concept of empowerment is particularly apt in the context of care, as it avoids a reductionist understanding of autonomy and takes into account personal and structural dependencies, power hierarchies, and individual restrictions and vulnerabilities [42]. In the context of dementia care, empowerment aims to strengthen the abilities of persons with dementia, involve them in decision-making and gaining control regarding their own lives, support them in creating changes that enlarge personal freedom, reduce stigma, and improve relationships and interactions [33, 34, 46, 51].

Some have argued that IAT could empower persons with dementia by a) supporting the quick and safe performance of daily activities that would otherwise require nursing support, b) expanding mobility and independence by minimizing the risk of accident inside and outside the home, and c) increasing privacy by replacing the need for human assistance with intimate interactions such as showering or toileting [26, 39, 42, 52]. Despite assistive technologies' empowering potential, critics point to the potential negative impact of IAT use on the empowerment of persons with dementia. IAT involve the risk of undermining the autonomy and privacy of persons with dementia [2, 7]. AI-driven autonomous technologies may undermine the goal of empowering persons with dementia because the autonomy of the system may conflict with the autonomy of the user [4, 7]. Improving privacy in intimate situations through IAT would require a continuous monitoring and collection of large amounts of data, which in turn compromises privacy [8]. Technologies can further create new dependencies and risks for users. If the interests, needs, and perspectives of users are not included in technology development at an early stage, technology design risks privileging the position of healthcare stakeholders, such as nurses, physicians, or technological industries, and might thus reproduce asymmetrical power relations [40, 42].

If one takes the perspective of empowerment theory seriously, it is not enough to focus only on the potential of IAT to increase autonomy and improve life quality. Instead, it must be better determined whether technologies are tailored to the specific needs, competencies, and interests of users, allowing users to employ them autonomously and in accordance with their interests and capabilities. To address these considerations, our study approaches empowerment from within a care ethics framework that emphasizes interpersonal relationships, care and context sensitivity [54]. This framework allows us to examine the impact of assistive technologies on the empowerment of persons with dementia, focusing on individual needs and the quality of the care relationship. Care ethics emphasizes the interdependence between persons with dementia and caregivers and recognizes that empowerment involves both autonomy and a supportive environment. It also allows us to examine the power relations that arise from using such technologies, particularly in relation to autonomy and privacy. Persons with dementia tend not to be sufficiently involved in the development process and evaluation of assistive technologies in dementia care; thus, the impact of IAT on everyday life and empowerment of persons with dementia remains unclear [31, 41, 52]. Furthermore, it is essential to involve both persons with dementia and caregivers in research together, as caregivers directly influence the experience and well-being of individuals through their use of IAT. This joint involvement could help identify differences and similarities in needs, opportunities, and risks to create solutions that enhance both the quality of life of the person with dementia and the support provided to caregivers [29, 44].

To address this gap, our study explores the perspectives of persons with dementia and their caregivers in Germany regarding the empowering potential of three exemplary intelligent assistive technologies: a GPS bracelet for independent outdoor mobility, an emotion recognition technology for detecting and managing negative emotions, and a dressing technology for independent dressing. We aim to examine IAT that address different fundamental aspects of life: daily functional activities, cognitive and emotional well-being, and interpersonal relationships. This approach allows us to capture a broader range of opportunities and risks and ensures that our findings do not reduce IATs to a single domain but rather highlight their diverse and context-specific implications. Based on interviews with persons with dementia and their caregivers, we investigate how IAT could impact different dimensions of everyday life (such as independence, safety, social participation, and privacy) and how practicable they are for everyday use. We aim to identify factors that promote and hinder an empowering application of IAT in accordance with users' needs and interests. In addition, we compare the perspectives of persons with dementia and caregivers regarding their envisioned use and potential impact of each of these technologies.

## Methods

### Study design

Our study used an exploratory, qualitative approach, aiming to understand the perspectives of the participants in the context of their everyday realities. We explored the attitudes, wishes and concerns of persons with dementia and their family caregivers regarding IAT in home care settings and nursing homes in Germany. Based on structured qualitative content analysis [27], semi-structured interviews (*n* = 27) with persons with dementia and family caregivers were analyzed to gain deeper insights into their perspectives on the impact of IAT on the empowerment of persons with dementia. An individualized interview guide was used for both interview groups, each containing three fictional case vignettes describing the function and use of three assistive technologies.

### Sampling, recruitment and consent

Participants were recruited between March 2020 and March 2022. Purposive sampling was used to recruit participants for the study, which is suitable for relatively small samples to select participants who are likely to provide relevant and useful information [10]. Participants were recruited by study staff and clinic staff in local hospitals and nursing homes. Participants were informed about the study by a research group member and by means of a participant information sheet. The study included 12 persons with dementia, aged 65–90 years (mean = 76.2), who met the criteria for a dementia diagnosis or had a Mini-Mental Status Examination score below 28. The participants were either patients at the German Centre for Neurodegenerative Diseases or patients at geriatric wards where an MMSE is routinely administered on admission. In the nursing homes or self-help groups, we interviewed participants who had been diagnosed with dementia according to their own information or that of the facility management. Except for one person with dementia with intermediate-stage dementia and one person with advanced-stage dementia, all participants with dementia had early-stage dementia. Interviews with persons with dementia were conducted in hospital (2), at home (5) or in a nursing home (5). Except for three persons with dementia who were interviewed in the presence of a relative or caregiver who did not take part in the interview, all persons with dementia were interviewed alone. The study included 15 family caregivers, aged 33–70 years (mean = 56.9), who were caring for a relative with dementia either at the time of data collection (*n* = 9) or in the past (*n* = 6). All but three caregivers were female (Table 1). Interviews with caregivers were conducted in hospital (7), at home (4), in a nursing home (1) or by telephone (3). In the case of a telephone interview, participants were given an information sheet with the case vignettes beforehand. All caregivers were interviewed alone.

**Table 1:** Participant characteristics (*n* = 27)

Pseudonym^a^	Age	Gender	Education Level/Profession	Care setting
Mr. Schulz (PwD) *(Husband of Ms. Krause)*	66	m	Housekeeper	Nursing home
Mr. Lang (PwD)	77	m	Secondary school/Farmer	Home care
Ms. Walter (PwD) *(Mother of Ms. Fischer)*	84	f	Pensioner	Nursing home
Mr. König (PwD) *(Husband of MS. Lehmann)*	81	m	University degree/engineer	Nursing home
Mr. Baumann (PwD)	91	m	Teacher	Home care
Ms. Berger (PwD)	83	f	Housekeeper	Home care
Ms. Roth (PwD)	81	f	Secondary school degree	Nursing home
Mr. Simon (PwD)	79	m	Secondary school degree, farmer	Nursing home
Ms. Wagner (PwD)	71	f	Import and export merchant	Nursing home
Mr. Schwarz (PwD)	53	m	Engineer	Home care
Ms. Lorenz (PwD)		f	Teacher	Nursing home
Mr. Braun (PwD)	78	m	Engineer	Home care
Ms. Schneider (CG)	67	f	University degree/teacher	Home care
Ms. Fischer (CG) *(Daughter of Ms. Walter)*	58	f	Geriatric nurse	Home care
Ms. Lehmann (CG) *(Wife of Mr. König)*	73	f	Preschool director	Home care
Ms. Weber (CG)	42	f		Home care
Ms. Krause (CG) *(Wife of Mr. Schulz)*	69	f	Pensioner	Home care
Ms. Neumann (CG)	81	f	University degree	Home care
Ms. Keller (CG)	42	f	University degree/Physician	Home care
Ms. Mayer (CG)	36	f		Home care
Ms. Vogel (CG)	52	f		Home care
Ms. Brandt (CG)	68	f	University degree	Home care
Mr. Beck (CG)	36	m	Administrative Assistant	Home care
Ms. Hofmann (CG)	73	f		Home care
Ms. Bauer (CG)	67	f	Administrative Assistant	Home care
Mr. Winkler (CG)		m		Home care
Mr. Hartmann (CG)		m	University degree	Home care

^a^*CG* Caregiver, *PwD* Person with Dementia. Information about participants who took part in the interviews as a pair, consisting of a caregiver and a person with dementia, is given in italics after the pseudonyms. All participants were interviewed independently

At the time of the data collection, approximately one-third of the respondents had experience with care technology. Five persons with dementia and three family caregivers used a simple home emergency system, and one family caregiver used an in-home motion sensor to monitor his wife. Approximately half of the respondents (*n* = 14) received support in everyday life from a professional care service at the time of data collection.

### Data collection

The data was collected as part of the joint project between March 2021 and March 2022 using semi-structured interviews with persons with dementia and informal caregivers. Separate interview guidelines for both interview groups were developed using the SPSS method of interview guideline development (see [17]). The interview guidelines were based on the central question and the project's interest in gaining insight into the perspectives of persons with dementia and their caregivers towards intelligent assistive technologies. The interview guidelines for the persons with dementia was adapted to their capabilities and needs by using simply worded interviews supplemented with explanatory visual material, using case vignettes. The case vignettes were developed in collaboration with an illustrator and focused on ensuring the comprehensibility and understandability of the technologies in concrete use scenarios. In addition, the interviews were conducted in the presence of a caregiver when necessary. These caregivers did not participate as interview partners.

The semi-structured interviews allow for responsiveness to interviewees’ focus areas while ensuring cross-case comparability. All interviews were conducted in German, and the quotations used in this paper have been translated into English. The interviews were transcribed according to previously established transcriptions guidelines [15] to ensure a verbatim and authentic reproduction of the interviews, including linguistic nuances and non-verbal elements. Care was taken to ensure a clear structure and to protect the confidentiality of the participants. For data analysis, the audio recordings and transcripts were pseudonymized and anonymized for publication. The average interview lasted approximately 42 min. Interviews with both groups began with open-ended questions focusing on daily routines, leisure activities, and the need for help with everyday tasks. Where appropriate, additional questions were added. The interview guidelines for informal caregivers starts with questions about the participants’ daily lives and caring activities. Questions were posed regarding caregivers’ definition of good care in the context of dementia and the current use of help and technical devices for the safety and health of the person being cared for. The interview guidelines for persons with dementia began with questions about various aspects of daily life and current living situation. First, persons with dementia were asked about the organization of daily life and activities that are important to them. This includes the role of their social environment and their own home. They were then asked about their support needs and current use of assistance technology. The introductory questions were followed by three case vignettes for each interview group, involving the three different technologies: Case vignette A: GPS bracelet; Case vignette B: Emotion recognition technology; Case vignette C: Dressing technology (Table 2).

**Table 2:** Overview of technologies analyzed

Technology	Description	Key feature
GPS bracelet	A wearable device that tracks the location of persons with dementia	*Version 1:* Provides continuous location updates *Version 2*: Alerts when the wearer exits a predefined area (geofencing)
Emotion recognition technology	A system that detects early signs of negative emotions using camera observations	Alerts caregivers; suggests appropriate interventions for persons with dementia and caregivers; no transmission of visual/audio material
Dressing Technology	A tool that assists individuals with dementia in dressing independently	*Version 1:* Smart clothes hanger that helps choose clothing order *Version 2:* Interactive system (called DRESS); communicate via a screen; helps choose the right clothes for the occasion

**Fig. 1:**
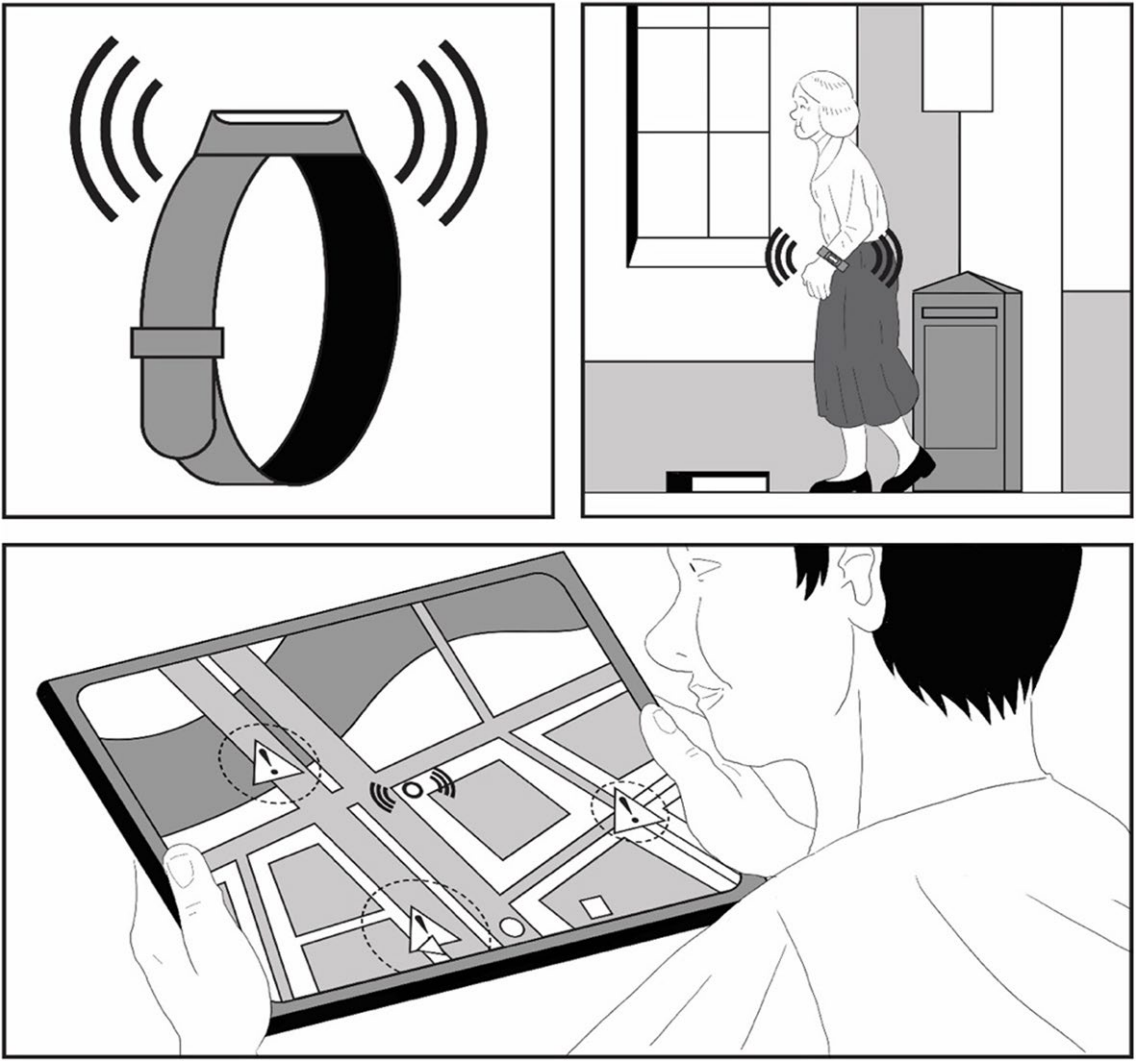
Case vignette A: GPS bracelet

By including a GPS bracelet, emotion recognition technology and a dressing device, the study aimed to capture a broad perspective on the different applications, functions and complexities of assistive technologies, and to identify challenges and opportunities for different user groups. The three selected technologies specifically cover different areas of life in the context of IAT, such as independence in daily activities, support for social participation and communication, and promotion of mental well-being. We included both passive applications (such as the GPS bracelet) and active applications (such as the dressing technology) to address the different needs and abilities of person with dementia. In addition, emotion recognition technology was included in the study to explore the extent to which assistive technologies can meet not only physical, but also psychological and communicative needs. By considering both functional and emotional aspects of IAT, the aim was to develop a more comprehensive understanding of their potential, encompassing both their practical support in everyday life and their impact on social interactions and emotional well-being. The aim was to reflect on the range of technological possibilities and to systematically identify potential challenges and opportunities for different user groups. Both groups were asked about the impact of the technologies on various desirable goals: independence, safety, privacy, social participation, and practicability in daily use. The case vignettes were used to illustrate the different technologies, explore the participants’ perspectives as realistically as possible, and better visualize the technologies’ impact. A pictorial representation in the form of a short comic strip complemented the case vignettes (See Fig. 1 and supplementary figures S1, S2 and S3 in the supplementary file 1).

### Data analysis

The transcribed interviews were analyzed using structural qualitative content analysis [27], a method particularly well-suited for semi-structured interviews with open research questions. This approach combines systematic organization and categorization of data with the flexibility to explore emerging themes, ensuring a comprehensive understanding of participants' perspectives. The thematic categories were formed deductively following a literature review of current research on the empowerment of persons with dementia [14, 42, 50], and the interview guidelines. New themes that emerged during the analysis process were supplemented by inductive categories. The interviews with persons with dementia and caregivers were coded separately for both interview groups using an individual coding guideline. The codes generated were paraphrased, generalized, and summarized into core statements by one researcher to extract and respond to relevant content from the collected interview data. The extracted content was analyzed using the following key terms: independence, safety, privacy, social participation, and practicability of the devices.

Established qualitative research criteria were applied to ensure the credibility of the findings [38] . Credibility was enhanced through researcher triangulation to reduce subjectivity and incorporate multiple perspectives. Peer coding was used, with two researchers analyzing 20% of the interviews to ensure consistency before one researcher recoded the entire dataset. Researchers Clara Löbe (medical doctor) and Niklas Peterson (sociologist) worked closely together in an interdisciplinary team of sociologists, medical ethicists and doctors. Methods, interpretations and findings were critically discussed in interdisciplinary meetings. In addition, the study context, participant demographics, and technologies analyzed were thoroughly documented, and the analysis process was transparently recorded.

## Results

The results of the qualitative content analysis of the interviews with persons with dementia and caregivers are presented in three sections: (1) first the results for the GPS bracelet, (2) followed by the results for the emotion recognition technology and (3) finally the results for the dressing technology.

### GPS bracelet

GPS devices were developed to locate mobile seniors with orientation problems outside their homes. The person in need of support wears a transmitting device, e.g., a GPS bracelet, which can send a signal with the current location, allowing relatives and caregivers to determine the location of this person. Some GPS bracelets can also give direct feedback to the person in need of support to find their way home. In our case vignette, interviewees were asked about two possible versions of the GPS bracelet. One device version could constantly send the persons with dementia's location to the caregiver, allowing the caregiver to determine the persons with dementia’s location permanently; the other version would only send a signal if the persons with dementia left a predefined area (*geofencing*).

Respondents saw the GPS bracelet as an opportunity to enhance the independence and mobility of persons with dementia, increase their safety, and reduce the burden on caregivers. Still, they also raised concerns about the risk of patronization and loss of privacy.

#### Independence

Interviewees mentioned different areas in which the GPS bracelet can strengthen the independence of persons with dementia, such as increased mobility, increased independence from others, more social participation, and longer stays in their home environment.

Respondents associate independence centrally with mobility. Users desire to be autonomous, for example, when shopping or taking walks. Respondents felt that the GPS wristband would be easy for persons with dementia to use, as they did not need any technical knowledge. Family caregivers hoped the GPS bracelet would help persons with dementia organize their daily lives as they wish, to be less dependent on help from relatives and caregivers. When asked how the GPS bracelet could promote the independence of persons with dementia, a family caregiver responded:

*”For me, if I had dementia and were still interested in just going shopping and doing things myself and not being dependent on having someone with me all the time […], that would be a good way to maintain my independence. The alternative would be that you would have to be accompanied in all your activities and wouldn’t be able to do anything on your own.” Ms. Schneider, family caregiver*

Persons with dementia shared this assumption and felt that the GPS bracelet would be particularly helpful for persons with advanced dementia who have difficulty orientating themselves in their familiar surroundings.

*“I could imagine it for people who have disorientation, who may have advanced Alzheimer's and so on. So sometimes I find myself looking for something.”* Mr. König, person with dementia

However, some respondents were concerned that the use of the GPS bracelet could also have a negative impact on the independence of persons with dementia. They were concerned that GPS bracelets could limit the freedom of choice of persons with dementia by prescribing routes or by immediately triggering an alarm in the event of spontaneous changes to familiar walking routes. Some caregivers also argued that monitoring every step of persons with dementia to avoid potential danger and allow persons with dementia to be more independent could become a burden for caregivers, as they would have to be constantly alert.

#### Safety

Interviewees also discussed the impact of the GPS bracelet on the safety of persons with dementia and caregivers. The hoped-for positive effects mentioned were: preventing dangerous situations in public spaces and increased emotional sense of safety, as persons with dementia would not worry as much about getting lost. Increased safety in public spaces was mentioned primarily by caregivers as a central argument for the use of the GPS bracelet, and some were willing to restrict the privacy of persons with dementia to guarantee their safety. They emphasized that an increased sense of safety could also lighten their care work, as they would not have to worry constantly. When asked how the GPS bracelet would affect their care work, one caregiver responded:

*“I think it's a very calming feeling for them and their loved ones when you can locate the person and see where they are.”* Ms. Bauer, family caregiver

Although some of the persons with dementia did not see a current need for GPS, e.g. because they had no orientation difficulties or were supported by relatives, they saw the technology as improving their safety in the event of progressive orientation difficulties.

*“If I were to order something like this, I would promise myself that I would use and apply it in an emergency. It would certainly improve my safety.”* Mr. Baumann, person with dementia.

However, interviewees also expressed concerns about the impact of using a GPS bracelet on the safety of persons with dementia, such as an increased risk of accidents outside the home. On the one hand, some persons with dementia rejected the increased independence offered by the GPS bracelet because they were afraid of accidents or getting lost. Some persons with dementia were willing to take this safety risk in favor of greater independence. Some family caregivers, however, pointed out that it would be irresponsible to send persons with dementia out on their own even with a GPS bracelet, as the technology could only be an aid but could not offset or reverse the effects of the disease.

#### Privacy

Most interviewees problematized the impact of GPS bracelets on privacy. Persons with dementia and caregivers noted the potential of users feeling patronized and about potential privacy violations. Only a few positive effects, related to privacy, were perceived, such as enabling mobility without direct personal supervision.

Permanent tracking through a GPS bracelet was vehemently rejected by many respondents, who argued that privacy must be guaranteed even for older adults or individuals with health conditions. Some respondents were concerned that the constant monitoring could patronize persons with dementia if it means they could not make normal decisions, such as where to shop or whom to visit, without other people being informed. Some persons with dementia even said they would be willing to accept lower levels of safety if it allowed them to maintain privacy. Asked if the technology would affect persons with dementia’s privacy, one person with dementia stated:

*“With this position tracker, it's like that: either he accepts it, or he doesn't. Done. But privacy…of course, you will always know when he goes somewhere he should not, and then he will certainly be reproached afterwards. That can't be avoided if someone is told they must not go to the lake but they keeping going there*.” Mr. Schwarz, person with dementia

In addition, family caregivers emphasized the importance of data security. Data access and processing should be transparent and well-protected.

Overall, GPS technology was viewed positively. Despite concerns regarding privacy and potential security risks, the technology was thought, overall, to promote independence, increase mobility, and improve safety for persons with dementia.

### Emotion recognition technology

Emotion recognition technologies are designed to detect potential negative emotions of persons with dementia, such as aggression, at an early stage and provide the opportunity for intervention to prevent conflict [16, 52]. The interviewees were asked about emotion recognition technology (ERT) that uses a camera to observe persons with dementia, informs caregivers when necessary, and suggests appropriate solutions for both the persons with dementia and their caregivers. During this process, the technology does not transmit visual or audio material.

When discussing the opportunities and risks of the emotion recognition technology, respondents were wary about constant monitoring and the risk of patronizing persons with dementia by controlling and suppressing their emotions. They also feared that technological monitoring instead of regular control by a human caregiver could lead to a lack of human interaction in care. Despite these strong concerns, some respondents see the potential to strengthen the relationship between persons with dementia and their caregivers by reducing conflicts and negative emotions.

#### Privacy

This form of emotion recognition technology is perceived mainly as disempowering, especially when persons with dementia may not have been asking for their consent. When asked about the impact of the emotion recognition technology on privacy, one person with dementia responded:

*“Yes, that would be like case one, a constant observation thing, at that point. Yes, of course, if someone sees that there's always a video camera on and can appreciate the meaning of it and has a feeling of being watched, that's not right, you know? Well…that's frustrating. I think it's a bit of a problem even for a normal person. So, when a camera (makes camera-like noises) tracks you, well, that's kind of/ you think, I'd rather go somewhere else or something, right?"* Mr. Schwarz, person with dementia

Caregivers argued that this could create an ethical conflict by hindering persons with dementia from expressing emotions. As a result, some persons with dementia, as well as some caregivers, felt that the technology was not in the best interests of the users. Persons with dementia also wanted to be able to express negative emotions, not just emotions desired by others. Due to that, some family caregivers feared a violation of rights. One family caregiver expressed her concerns as follows:

*“They might not notice, of course, but the bottom line is that you're being filmed all the time. In my opinion… if the question of data protection security was an issue with a tracking bracelet, the issue is even stronger if grandma is sitting on the sofa and being filmed the whole time.… Even I as a…caring relative would probably have a problem with data protection or with protecting the person’s personal rights."* Mr. Beck, family caregiver

One caregiver also noted that constant monitoring could change caregivers’ work as they would also be aware of the surveillance. Caregivers had different opinions about how technology would affect persons with dementia and caregivers’ privacy in private or professional care settings. Some argued that persons with dementia lose some privacy in a nursing home in any case. In contrast, others felt that consent to technology would be possible only in a private setting, as there would be too many people in a nursing home.

#### Social participation

Some caregivers and persons with dementia hoped that emotion recognition technology could improve the relationship between persons with dementia and caregivers and between persons with dementia and non-caregivers if timely recognition of bad moods and targeted responses could prevent negative emotions and associated conflicts. When asked about the impact of technology on her daily work, one family caregiver responded:

*“Again, that's a quality-of-life issue for me. If I get into an aggressive dispute, it’s not over after a ten-minute argument. It continues to have an effect. And why do I have to deal with such situations when there's a technical solution that warns me or prepares me for something. And perhaps even shows me what possibility of help…there is. I think it’s good.”* Ms. Lehmann, family caregiver

Many respondents considered the impact of the emotion recognition technology on persons with dementia and caregivers’ relationships and social life to be problematic. Many caregivers and persons with dementia criticized the lack of human interaction in technology-based care. They feared that the relationship between persons with dementia and caregivers could be strained if persons with dementia experienced a loss of trust due to the awareness of monitoring. Some respondents were concerned that caregivers could exercise power over persons with dementia, controlling their emotions to achieve more socially acceptable behavior. They feared that if persons with dementia live with the awareness of being constantly monitored, this could have unwanted effects, such as suppressing emotions altogether. When asked about the impact of technology on persons with dementia's experience of emotions, one caregiver responded:

*“Yes, and if someone were to take [emotion] away from you, permanently, always, at the first onset…immediately someone comes and tries to calm you down, how terrible…. Then you might as well put someone on drugs and say, well, so you're now on your happiness meds and can…stay in the same mood…. I think…aggression is just part of life, too."* Ms. Schneider, family caregiver.

The view that persons with dementia have a right to act out their emotions freely if they do not endanger anyone was emphasized by many caregivers and persons with dementia. Moreover, it was feared that the constant monitoring of the emotions of persons with dementia could increase anxiety and worry among family caregivers.

Overall, the technology was evaluated rather critically. Although some persons with dementia and caregivers acknowledge that the use of the technology could avoid conflicts, most interviewees fear a violation of the privacy of persons with dementia and caregivers, suppression, control of emotions, and reduced human support.

### Dressing technology

Dressing technologies are designed to help persons with dementia dress independently. Smart clothes hangers are designed to help a person with dementia dress in the right order. An extension of the system, called DRESS, can communicate with a screen, and make suggestions for clothing depending on, for example, the occasion or the weather [9, 32].

Persons with dementia and caregivers expressed critical views of dressing technology, especially the smart clothes hanger, doubting it could accommodate user interests or be practicable. They also feared a reduction of human interaction in care. Despite these critical opinions, some persons with dementia and caregivers believed that dressing technology could provide an opportunity to increase the social interaction of persons with dementia, increase their self-confidence, protect them from stigmatization, and reduce their dependence on others.

#### Independence

Some caregivers and persons with dementia said that dressing technology could increase the independence of persons with dementia by helping them dress on their own. When asked if it makes a difference to persons with dementia whether they take dressing suggestions from technology or a caregiver, one family caregiver responded:

*“Yes. If the hanger tells him, he'll take that more readily than if the person caring for him tells him and puts it out for him. If he was always used to getting things out himself before, […] it is sometimes difficult to be dependent on other people to put things out for you. You then very quickly feel patronized.”* Ms. Schneider, family caregiver

Some respondents were optimistic that the dressing technology could help persons with dementia gain decision-making authority about when and how they dress. However, respondents noted that there can be a fine line between empowerment and patronization. Many respondents rejected prescriptive instructions or clothing choices by technology that could not be altered. They were concerned that IAT dictating clothing choices to persons with dementia could be patronizing and make persons with dementia dependent on technology. Still, some respondents found it acceptable if the technology only suggests the dressing sequence.

#### Social participation 

Persons with dementia and caregivers both expressed that dressing technology could increase social participation and acceptance of persons with dementia if they did not have to worry about being inappropriately dressed. When asked about the impact of technology on the social participation of persons with dementia, one family caregiver responded:

*“Yeah, I think that's where…it can have a positive impact, if he can manage the order of how you dress with technological support, that will probably increase his sense of well-being…and the acceptance in his environment will also increase.”* Ms. Bauer, family caregiver

Most of the interviewees saw the potential avoidance of stigma as positive. Some hoped this could also strengthen persons with dementia’s self-esteem if it made them feel better about themselves. In addition, some caregivers hoped they would have to worry less about the person in their care being inappropriately dressed.

Despite the anticipated benefits of the technology, interviewees' apprehensions were palpable. The absence of human interaction in care was a recurring concern. In the interviews, participants with dementia preferred human support, despite the potential for increased independence through dressing technology. When asked if he could envision using a dressing technology, one person with dementia responded:

*“I don't see it personally so far…. I can't imagine that it would be a huge win for me…. I can imagine that a person who takes care of me would consider it a win, but a robot would be nothing for me. That's a toy for me, so to say.”* Mr. Braun, person with dementia

This strong inclination to uphold human interaction in care was evident among individuals with dementia and shared by caregivers [18, 51].

#### Practicability

Most respondents expressed skepticism about the practical application of dressing technology. They cited the technology's lack of adaptation to users’ capabilities as a critical barrier. The following detailed comment about practical concerns provides insight into the challenges and limitations of IAT in dementia care.

*“I think it's interesting that Mr. M. still goes to the theater alone, apparently, but he doesn't dress anymore […] I can only say that now, when I see my mother, it wouldn't matter at all if the hangers were hanging there. If she doesn't feel like getting dressed, or maybe she has already forgotten where I'm going. So, that already presupposes a certain basic independence. He knows he wants to go there and to go there today; he knows when he must get dressed and ready and then he gets dressed in time…. [S]o it wouldn't work for my mother, she would still just put on her undershirt and underpants and probably go back to bed because she thinks she still has five hours."* Ms. Mayer, family caregiver

Most interviewees shared the assessment that persons with dementia who are cognitively unable to dress independently would still be unable to attend events alone. Some persons with dementia particularly disliked the version of the dressing technology that communicates via a screen (DRESS System), as they found the technology frightening and overwhelming. Some of the caregivers shared this concern, noting that persons with dementia may not have sufficient technology skills or may not be used to using technology to effectively benefit from the dressing technology.

Despite its potential benefits, the concerns raised by persons with dementia and caregivers about the dressing technology are significant. These include the lack of applicability in daily use, the failure to adapt to the needs and abilities of the potential user group, and the absence of human interaction in care.

## Discussion

The study shows that persons with dementia and their caregivers in Germany believe that IAT could have the potential to empower persons with dementia by improving their independence in everyday tasks, supporting their independent mobility, increasing their physical safety and sense of emotional security, and improving their participation in social interactions. However, persons with dementia and caregivers also raised concerns about the use of IAT, around invasion of privacy, patronization of persons with dementia, lack of human interaction in care, risk of self-harm in the absence of caregivers, and lack of adaptation of the technology to the interests and abilities of persons with dementia.

Although most participants with dementia were not using the technologies discussed at the time of data collection, and their perspectives were based on hypothetical use rather than personal experience, they were still able to develop clear attitudes towards their potential applications. Many persons with dementia refused to use the technologies described in the fictional case vignettes in real life at the time of data collection. However, persons with dementia could imagine using the technologies if their disease progressed, and they imagined that some technologies might be useful for others with severe dementia symptoms. The results showed that persons with dementia, despite limited experience, were able to form opinions about technologies. This is a valuable insight, demonstrating that persons with dementia can be seen not only as passive subjects, but also as active partners in reflection in research. The findings highlight that participatory research is essential if new technologies are to be developed in line with the empowerment approach, incorporating users' needs, preferences and perspectives into the design and development process. Similar conclusions have been reached in other studies, which emphasize the importance of involving persons with dementia and caregivers in co-design processes to ensure that technologies are both practical and consistent with their lived experiences and values [5, 19, 29]. These studies highlight that meaningful participation not only promotes better usability, but also greater acceptance and relevance of assistive technologies in dementia care.

Overall, persons with dementia and caregivers do not express unified approval or disapproval of IAT in dementia care in general. Instead, their evaluation of the technologies seems to depend on various factors, such as their impact on safety, privacy, social participation, or independence or on the technologies' practicability in daily use in accordance with user interest (see Table 3). Although both persons with dementia and caregivers showed similar trends in their evaluations, significant differences emerged, particularly in relation to safety and privacy. These findings highlight that evaluation results from studies assessing the applicability or impact of individual technologies on specific aspects of life cannot simply be generalized to other technologies or user groups. This underlines the need to integrate the empowerment approach into research to ensure that the diverse needs and perspectives of different user groups are adequately addressed.

**Table 3:** Assessments of IAT

	GPS Bracelet	Emotion Recognition Technology	Dressing Technology
**Independence**	+ increased mobility + increased autonomy in daily activities + strengthening of persons with dementia self-confidence - restricting freedom of movement - increased mobility of persons with dementia as a burden for caregivers *(CG)* - preference for security/human contact over independence *(PwD)*	- feeling of paternalism in expressing emotions - restriction of personal rights	+ increased independence in performing daily activities + decision-making authority about when and how to dress - creating new dependencies on technology - sense of fear *(PwD)* - prioritizing human interaction over autonomy *(PwD)*
**Safety**	+ emotional sense of safety + avoidance of getting lost - prioritizing of independence over safety *(PwD)* - willingness to limit PwD’s privacy for security *(CG)*	+ strengthened sense of security + prevention of violence against caregivers - Risk of self-harm in the case of absent caregivers	
**Social Participation**	+ feeling of more social participation	+ avoiding aggression and conflict + improved mood of persons with dementia - repression and control of emotions - unequal power relations by controlling the social behavior of persons with dementia - lack of human interaction - increased anxiety among family caregivers *(CG)*	+ avoidance of stigma + sense of more social participation - lack of human interaction *(PwD)* - fear of social isolation of PwD *(CG)*
**Privacy**	+ mobility without personal supervision - feeling of surveillance - data security concerns *(CG)*	- feeling of surveillance - technological monitoring in private living space - data security concerns *(CG)* - feeling of control by caregivers - monitoring care work *(CG)*	+ increased privacy during dressing and undressing
**Practicability**	+ simple handling in daily use + meets the user's wish for independent mobility - constant availability of caregivers required *(CG)*	- no adequate response to emotional needs - lack of orientation towards users' interests in expressing emotions	- not adapted to persons with dementia capabilities *(CG)* - no practical applicability in daily use

Differences in the assessment of the technologies by person with dementia (PwD) and family caregiver (CG) are indicated in the table by a corresponding abbreviation in bracket after the finding

### Independence

From the perspective of persons with dementia and their family caregivers, the claim that IAT could support the autonomy of persons with dementia and thus empower them is not supported for all technologies. Overall, technologies that can be used passively and without technical knowledge, such as the GPS bracelet, are seen to be more helpful in promoting persons with dementia’s independence, a finding that is consistent with the results of participatory dementia research on the use of IAT [44]. Technologies that require active use, such as the dressing technology, are considered less useful because of concerns that they could be beyond the capabilities of persons with dementia. Some persons with dementia found it difficult to envision the relevance of assistive technologies in their own lives, especially when they did not see any challenges in the described areas of use. This suggests that the perceived usefulness of a technology is closely linked to its alignment with the current needs and realities of the user's life. A clear understanding of how a technology might be useful to oneself appears to be a crucial prerequisite for the willingness to engage with it in the first place, a factor that also received attention in the existing literature [24, 55]. A lack of user specificity and everyday practicability of some technologies, especially the dressing technology, was one of the main criticisms in the interviews, a finding that is also reflected in other studies on the usability of IAT for dementia care [1, 2, 40]. Further, our study confirms worries that using IAT can create new dependencies, e.g. if persons with dementia are dependent on the help of a dressing technology without being able to influence functions such as clothing choice options. The findings highlight the ethical ambivalence of IATs, as they can simultaneously promote autonomy and potentially reinforce power asymmetries between caregivers and care recipients. The reproduction of power imbalances through IAT could be seen as a contradiction to the empowerment approach, which seeks to reduce power hierarchies and, at the same time, strengthen the decision-making authority of persons with dementia [42, 50].

### Safety

Promoting the safety of persons with dementia and caregivers is mentioned as an essential requirement for the successful use of IAT by both interview groups [25, 36, 40]. Some fear that transferring responsibility from human caregivers to technology could increase risks such as accidents or getting lost. However, persons with dementia and caregivers have different views about the importance of safety in the daily lives of persons with dementia. Some persons with dementia were generally opposed to increased independence through IAT because they feared an increased risk of accidents and would instead rather rely on the help of others rather than be independent but alone. Other persons with dementia emphasized that they would prefer to gain more independence by using IAT, even at the risk of lowering safety levels. In contrast, many caregivers were willing to limit the independence and privacy of persons with dementia to secure their safety. The tension between safety and autonomy contradicts paternalistic assumptions that safety should always be prioritized and highlights the importance of the empowerment approach in the context of care and IAT [37]. The differences in the evaluation of IAT usage also highlight the importance of informed consent when persons with dementia and caregivers use IAT. Persons with advanced dementia may no longer be able to provide informed consent due to cognitive impairment [12, 22]. This may cause a conflict with the goal of empowerment, which involves free will and keeping control over one's own life [11, 50].

### Privacy

A significant criticism of using IAT is the potential impact on persons with dementia and caregivers’ privacy, which both interview groups stressed. Confirming results of earlier studies [2, 4, 40, 43], respondents feel that IAT could create a sense of patronization and restriction of personal rights and lead to surveillance of persons with dementia and caregivers’ work. For some respondents, the presence of IAT alone is associated with a feeling of being surveilled, regardless of the actual use or function of the technology. This finding highlights the importance of considering the psychological impact of new technologies alongside their practical applications. Although the impact of IAT on privacy was viewed critically by both interview groups, our study shows that persons with dementia and caregivers viewed this impact differently in some respects. In their evaluation of the three technologies, persons with dementia, in general, rated privacy highly. In contrast, some caregivers were willing to restrict privacy. As caregivers and persons with dementia may both be users of an IAT but with different intentions, the question arises as to how the different interests of the user groups can be balanced. Other caregivers were concerned that the use of the IAT could lead to a possible invasion of the privacy of persons with dementia, but also of family members and caregivers by monitoring their care activities. This aspect seems particularly relevant to the empowerment approach, as it raises the question of whether caregivers who are weakened in their empowerment can contribute inadequately to the empowerment of the person with dementia [13, 56].

### Social participation

In our study, both persons with dementia and caregivers feared that the use of IAT might reduce interpersonal interaction, underlining the central role of the social context in the acceptability of IAT. These findings confirm the results of other studies observing concerns about social isolation and control through IAT in dementia care [2, 20]. Evoking positive emotions and caregivers' involvement are critical factors for the successful implementation of IAT [1]. Therefore, the strain on interpersonal relations could be a barrier to the successful implementation of IAT. Controlling the actions and social behavior of persons with dementia through IAT could intensify unequal power relations and thus counter the empowerment approach, which seeks to reduce power imbalances in care [42]. Our study shows that persons with dementia tend to prefer human interaction in care over greater autonomy. Furthermore, the findings point to the risk of pathologizing negative emotions through emotion recognition technologies, a perspective that has received limited attention. This concern, raised by both persons with dementia and caregivers, highlights the need for a nuanced understanding of emotional expression as a natural and valid part of the care process [28]. IAT interventions that aim to avert negative emotions or abandon social contact in care rather than considering the interests of the user, can therefore be seen as potentially weakening the empowerment of persons with dementia. In addition, some caregivers suggested that they would be more worried and anxious about their loved ones if they did activities on their own that they would not do without the support of the IAT. This raises the question of whether increased worry among caregivers might increase control over persons with dementia rather than increasing social participation or independent action by persons with dementia. In the interviews, IATs that promote socially appropriate behavior, such as choosing appropriate clothing, were seen primarily as helping to avoid stigmatizing persons with dementia. This contrasts with some criticism of IAT in research, which is concerned that restoring functionality may reproduce a discriminatory view of normality [45].

### Limitations

Although the findings of this study provide valuable insights into the perspectives of persons with dementia and their caregivers in relation to IAT, certain limitations should be considered to contextualize the findings. The relatively small sample size of 27 interviews may not fully capture the diversity of perspectives of persons with dementia and caregivers, which may limit the generalizability of the findings. In addition, only persons with early or intermediate dementia were included. The focus on three technologies further limits the scope, restricting broader conclusions on the potential of other assistive technologies. Despite these limitations, the study makes an important contribution to understanding the perception and evaluation of IAT by persons with dementia and their caregivers and provides a basis for future research and development of assistive technologies in dementia care.

## Conclusions

IAT has the potential to empower persons with dementia by promoting independence, supporting mobility, enhancing safety and improving social participation. However, our findings show that perceptions of the opportunities and perils of the three technologies varied widely, with concerns raised about invasion of privacy, patronization, reduced human interaction and lack of technological adaptability. Persons with dementia with no practical experience of IAT expressed difficulty in imagining how these technologies would fit into their lives, especially as their condition progressed. This may indicate that IAT is not sufficiently focused on the needs and interests of persons with dementia. Overall, the study highlights that there is no clear approval or disapproval position on the impact of using IAT to empower persons with dementia in general. Rather, the results of our study emphasize that its potential to empower persons with dementia depends largely on how well it is tailored to their individual needs and preferences. Studies investigating IAT in dementia care often confront persons with dementia with existing technologies, rather than exploring user needs before development begins. Further studies using a participatory approach to technology, which includes the needs, capabilities and wishes of user groups in the development and design process from the outset, could enable the development of technologies that better serve the interests of users and thus might effectively contribute to the empowerment of persons with dementia.

## Supplementary Information

Supplementary Material 1.

Supplementary Material 2.

Supplementary Material 3.

## Data Availability

The data from this study is not publicly available for reasons of date protection. Access to the encrypted versions of the material is restricted to the scientists involved in the research project. The empirical data collected are stored in a trusted cloud of the Gesellschaft für wissenschaftliche Datenverarbeitung mbH Göttingen (GWDG) "ownCloud".

## References

[1] Arntzen C, Holthe T, Jentoft R. Tracing the successful incorporation of assistive technology into everyday life for younger people with dementia and family carers. Dementia. 2016;15(4):646–62. 10.1177/1471301214532263. 24784941 10.1177/1471301214532263

[2] Asghar I, Cang S, Yu H. Usability evaluation of assistive technologies through qualitative research focusing on people with mild dementia. Comput Hum Behav. 2018;79:192–201. 10.1016/j.chb.2017.08.034.

[3] Aujoulat I, Marcolongo R, Bonadiman L, Deccache A. Reconsidering patient empowerment in chronic illness: a critique of models of self-efficacy and bodily control. Soc Sci Med. 2008;66(5):1228–39. 10.1016/j.socscimed.2007.11.034. 18155338 10.1016/j.socscimed.2007.11.034

[4] Bennett B, McDonald F, Beattie E, Carney T, Freckelton I, White B, Willmott L. Assistive technologies for people with dementia: Ethical considerations. Bull World Health Organ. 2017;95(11):749–55. 10.2471/BLT.16.187484. 29147055 10.2471/BLT.16.187484 PMC5677608

[5] Berridge C, Turner NR, Liu L, Karras SW, Chen A, Fredriksen-Goldsen K, Demiris G. Advance planning for technology use in dementia care: development, design, and feasibility of a novel self-administered decision-making tool. JMIR Aging. 2022;5(3). 10.2196/39335. 10.2196/39335 PMC9377442 35896014

[6] Brookman R, Parker S, Hoon L, Ono A, Fukayama A, Matsukawa H, Harris CB. Technology for dementia care: what would good technology look like and do, from carers’ perspectives? BMC Geriatr. 2023;23(1):867. 10.1186/s12877-023-04530-9. 38104074 10.1186/s12877-023-04530-9 PMC10725604

[7] Buhr E, Schweda M. Der Wert des Privaten für Menschen mit Demenz. Ethik in der Medizin. 2022;34(4):591–607. 10.1007/s00481-022-00723-9.

[8] Buhr, E., & Welsch, J. (Eds.). (2022). Privacy-sensitive Empowerment.1 Towards an integrated ethical concept for technology-assisted care for people with dementia (1st ed.). V&R unipress. 10.14220/9783737014793.

[9] Burleson W, Lozano C, Ravishankar V, Lee J, Mahoney D. An assistive technology system that provides personalized dressing support for people living with dementia: capability study. JMIR Med Inform. 2018;6(2):321–35. 10.2196/medinform.5587. 10.2196/medinform.5587 PMC5954231 29716885

[10] Campbell S, Greenwood M, Prior S, Shearer T, Walkem K, Young S, Bywaters D, Walker K. Purposive sampling: complex or simple? Research case examples. J Res Nurs. 2020;25(8):652–61. 10.1177/1744987120927206. 34394687 10.1177/1744987120927206 PMC7932468

[11] Castro EM, Van Regenmortel T, Vanhaecht K, Sermeus W, Van Hecke A. Patient empowerment, patient participation and patient-centeredness in hospital care: a concept analysis based on a literature review. Patient Educ Couns. 2016;99(12):1923–39. 10.1016/j.pec.2016.07.026. 27450481 10.1016/j.pec.2016.07.026

[12] Cunningham E, McGuinness B, Herron B, Passmore A. Dementia. Ulster Med J. 2015;84(2):79–87. 26170481 PMC4488926

[13] Deyhoul N, Vasli P, Rohani C, Shakeri N, Hosseini M. The effect of family-centered empowerment program on the family caregiver burden and the activities of daily living of Iranian patients with stroke: a randomized controlled trial study. Aging Clin Exp Res. 2020;32(7):1343–52. 10.1007/s40520-019-01321-4. 31473982 10.1007/s40520-019-01321-4

[14] Feste C, Anderson RM. Empowerment: from philosophy to practice. Patient Educ Couns. 1995;26(1–3):139–44. 10.1016/0738-3991(95)00730-N. 7494713 10.1016/0738-3991(95)00730-n

[15] Fuß S, Karbach U. Grundlagen der Transkription. In Grundlagen der Transkription (2., pp. 1–11). Verlag Barbara Budrich. 2019. https://www.utb.de/doi/abs/10.36198/9783838550749-1-11.

[16] Hartmann KV, Rubeis G, Primc N. Healthy and happy? An ethical investigation of emotion recognition and regulation technologies (ERR) within ambient assisted living (AAL). Sci Eng Ethics. 2024;30(1):2. 10.1007/s11948-024-00470-8. 38270734 10.1007/s11948-024-00470-8 PMC10811057

[17] Helfferich C. Die Qualität qualitativer Daten. VS Verlag für Sozialwissenschaften. 2011. 10.1007/978-3-531-92076-4.

[18] Holmström I, Röing M. The relation between patient-centeredness and patient empowerment: a discussion on concepts. Patient Educ Couns. 2010;79(2):167–72. 10.1016/j.pec.2009.08.008. 19748203 10.1016/j.pec.2009.08.008

[19] Huang H-H, Chang M-H, Chen P-T, Lin C-L, Sung P-S, Chen C-H, Fan S-Y. Exploring factors affecting the acceptance of fall detection technology among older adults and their families: a content analysis. BMC Geriatrics. 2024;24(1), Article 1. 10.1186/s12877-024-05262-0. 10.1186/s12877-024-05262-0 PMC11334405 39164655

[20] Ienca M, Wangmo T, Jotterand F, Kressig RW, Elger B. Ethical design of intelligent assistive technologies for dementia: a descriptive review. Sci Eng Ethics. 2018;24(4):1035–55. 10.1007/s11948-017-9976-1. 28940133 10.1007/s11948-017-9976-1

[21] Kenigsberg P-A, Aquino J-P, Berard A, Bremond F, Charras K, Dening T, Droes R-M, Gzil F, Hicks B, Innes A, Nguyen S-M, Nygard L, Pino M, Sacco G, Salmon E, van der Roest H, Villet H, Villez M, Robert P, Manera V. Assistive technologies to address capabilities of people with dementia: from research to practice. Dementia (London, England). 2019;18(4):1568–95. 10.1177/1471301217714093. 28699364 10.1177/1471301217714093

[22] Kim B, Noh GO, Kim K. Behavioural and psychological symptoms of dementia in patients with Alzheimer’s disease and family caregiver burden: a path analysis. BMC Geriatr. 2021;21(1):160. 10.1186/s12877-021-02109-w. 33663416 10.1186/s12877-021-02109-w PMC7934246

[23] Köhler S, Perry J, Biernetzky OA, Kirste T, Teipel SJ. Ethics, design, and implementation criteria of digital assistive technologies for people with dementia from a multiple stakeholder perspective: a qualitative study. BMC Med Ethics. 2024;25(1):84. 10.1186/s12910-024-01080-6. 39068472 10.1186/s12910-024-01080-6 PMC11282641

[24] König T, Pigliautile M, Águila O, Arambarri J, Christophorou C, Colombo M, Constantinides A, Curia R, Dankl K, Hanke S, Mayer CC, Moritsch S, Müllner-Rieder M, Pernkopf F, Schüler C, Stillo M, Mecocci P, Stögmann E. User experience and acceptance of a device assisting persons with dementia in daily life: a multicenter field study. Aging Clin Exp Res. 2022;34(4):869–79. 10.1007/s40520-021-02013-8. 34762252 10.1007/s40520-021-02013-8 PMC8581127

[25] Kowe A, Köhler S, Görß D, Teipel S. The patients’ and caregivers’ perspective: In-hospital navigation aids for people with dementia- a qualitative study with a value sensitive design approach. Assist Technol. 2023;35(3):248–57. 10.1080/10400435.2021.2020378. 34919023 10.1080/10400435.2021.2020378

[26] Kruse CS, Fohn J, Umunnakwe G, Patel K, Patel S. Evaluating the facilitators, barriers, and medical outcomes commensurate with the use of assistive technology to support people with dementia: a systematic review literature. Healthcare. 2020;8(3):278. 10.3390/healthcare8030278. 32824711 10.3390/healthcare8030278 PMC7551699

[27] Kuckartz U. Qualitative text analysis: a guide to methods. Practice and Using Software. London: SAGE Publications Ltd; 2014.

[28] Lee KH, Lee JY, Boltz M, McConnell ES. Emotional expression of persons with dementia: an integrative review with implications for evidence-based practice. Worldviews Evid Based Nurs. 2019;16(5):344–51. 10.1111/wvn.12395. 31397542 10.1111/wvn.12395

[29] Liddle J, Worthy P, Frost D, Taylor E, Taylor D. Partnering with people living with dementia and care partners in technology research and design: reflections and recommendations. Aust Occup Ther J. 2022;69(6):723–41. 10.1111/1440-1630.12843. 36203322 10.1111/1440-1630.12843 PMC10092369

[30] Liu L, Miguel Cruz A, Ruptash T, Barnard S, Juzwishin D. Acceptance of global positioning system (GPS) technology among dementia clients and family caregivers. J Technol Hum Serv. 2017;35(2):99–119. 10.1080/15228835.2016.1266724.

[31] Löbe C, AboJabel H. Empowering people with dementia via using intelligent assistive technology: a scoping review. Arch Gerontol Geriatr. 2022;101:104699. 10.1016/j.archger.2022.104699. 35413610 10.1016/j.archger.2022.104699

[32] Mahoney DF, Burleson W, Lozano C, Ravishankar V, Mahoney EL. Prototype development of a responsive emotive sensing system (DRESS) to aid older persons with dementia to dress independently. Gerontechnology. 2015;13(3):345–58. 10.4017/gt.2015.13.3.005.00. 26321895 10.4017/gt.2015.13.3.005.00 PMC4551505

[33] McConnell T, Best P, Sturm T, Stevenson M, Donnelly M, Taylor BJ, McCorry N. A translational case study of empowerment into practice: a realist evaluation of a member-led dementia empowerment service. Dementia (London, England). 2020;19(6):1974–96. 10.1177/1471301218814393. 30470153 10.1177/1471301218814393

[34] McConnell T, Sturm T, Stevenson M, McCorry N, Donnelly M, Taylor BJ, Best P. Co-producing a shared understanding and definition of empowerment with people with dementia. Res Involv Engagem. 2019;5:19. 10.1186/s40900-019-0154-2. 31205750 10.1186/s40900-019-0154-2 PMC6558688

[35] Meiland FJM, Bouman AIE, Sävenstedt S, Bentvelzen S, Davies RJ, Mulvenna MD, Nugent CD, Moelaert F, Hettinga ME, Bengtsson JE, Dröes R-M. Usability of a new electronic assistive device for community-dwelling persons with mild dementia. Aging Ment Health. 2012;16(5):584–91. 10.1080/13607863.2011.651433. 22360649 10.1080/13607863.2011.651433

[36] Meiland FJM, Hattink BJJ, Overmars-Marx T, de Boer ME, Jedlitschka A, Ebben PWG, Stalpers-Croeze IINW, Flick S, van der Leeuw J, Karkowski IP, Dröes RM. Participation of end users in the design of assistive technology for people with mild to severe cognitive problems; the European Rosetta project. Int Psychogeriatr. 2014;26(5):769–79. 10.1017/S1041610214000088. 24507571 10.1017/S1041610214000088

[37] Moermans V, Bleijlevens M, Verbeek H, Milisen K, de Casterlé BD, Hamers J. Safety or autonomy in dementia care at home, a qualitative study on family caregivers’ experiences. Innov Aging. 2020;4(Suppl 1):435. 10.1093/geroni/igaa057.1406.

[38] Moser A, Korstjens I. Series: Practical guidance to qualitative research. Part 3: Sampling, data collection and analysis. Eur J Gen Pract. 2017;24(1): 9–18. 10.1080/13814788.2017.1375091. 10.1080/13814788.2017.1375091 PMC5774281 29199486

[39] Moyle W. The promise of technology in the future of dementia care. Nature Reviews Neurology. 2019;15(6), Article 6. 10.1038/s41582-019-0188-y. 10.1038/s41582-019-0188-y 31073242

[40] Olsson A, Skovdahl K, Engström M. Using diffusion of innovation theory to describe perceptions of a passive positioning alarm among persons with mild dementia: a repeated interview study. BMC Geriatr. 2016;16:3. 10.1186/s12877-016-0183-8. 26745961 10.1186/s12877-016-0183-8 PMC4706660

[41] Rai HK, Cavalcanti Barroso A, Yates L, Schneider J, Orrell M. Involvement of People with dementia in the development of technology-based interventions: narrative synthesis review and best practice guidelines. J Med Internet Res. 2020;22(12):e17531. 10.2196/17531. 33270034 10.2196/17531 PMC7746489

[42] Schicktanz S, Schweda M. Aging 4.0? Rethinking the ethical framing of technology-assisted eldercare. Hist Philos Life Sci. 2021;43(3):93. 10.1007/s40656-021-00447-x. 34342739 10.1007/s40656-021-00447-x PMC8332600

[43] Schweda M, Kirste, Thomas, Hein, Andreas, Teipel, Stefan, Schicktanz, Silke. The emergence of co-intelligent monitoring and assistive technologies in dementia care – an outline of technological trends and ethical aspects. Bioethica Forum. 2020;12:29–37. 10.24894/BF.2019.12008.

[44] Shastri K, Boger J, Marashi S, Astell A, Dove E, Nedlund A-C, Mäki-Petäjä-Leinonen A, Nygård L. Working towards inclusion: creating technology for and with people living with mild cognitive impairment or dementia who are employed. Dementia. 2022;21(2):556–78. 10.1177/14713012211051885. 34749536 10.1177/14713012211051885 PMC8811323

[45] Silvers A. Better Than New! Ethics for Assistive Technologists. In Design and Use of Assistive Technology: Social, Technical, Ethical, and Economic Challenges (pp. 3–15). Springer. 2010. 10.1007/978-1-4419-7031-2_1.

[46] Smith R, Ooms A, Greenwood N. Supporting people with young onset dementia and their families: an evaluation of a training course for care workers. Nurse Educ Pract. 2017;27:7–12. 10.1016/j.nepr.2017.08.007. 28806593 10.1016/j.nepr.2017.08.007

[47] Sriram V. Carers’ experiences of assistive technology use in dementia care: a cross sectional survey. 2021:471. 10.1186/s12877-021-02417-1. 10.1186/s12877-021-02417-1 PMC8385483 34433416

[48] Suijkerbuijk S, Nap HH, Cornelisse L, IJsselsteijn WA, de Kort YAW, Minkman MMN. Active involvement of people with dementia: a systematic review of studies developing supportive technologies. J Alzheimer’s Dis. 2019;69(4):1041–65. 10.3233/JAD-190050. 10.3233/JAD-190050 PMC6597993 31156158

[49] Tiersen F, Batey P, Harrison MJC, Naar L, Serban A-I, Daniels SJC, Calvo RA. Smart home sensing and monitoring in households with dementia: user-centered design approach. JMIR Aging. 2021;4(3):e27047. 10.2196/27047. 34383672 10.2196/27047 PMC8387885

[50] van Corven C, Bielderman A, Wijnen M, Leontjevas R, Lucassen PL, Graff MJ, Gerritsen DL. Promoting empowerment for people living with dementia in nursing homes: development and feasibility evaluation of an empowerment program. Dementia. 2022;21(8):2517–35. 10.1177/14713012221124985. 36063815 10.1177/14713012221124985 PMC9583290

[51] Van Corven CTM, Bielderman A, Wijnen M, Leontjevas R, Lucassen PLBJ, Graff MJL, Gerritsen DL. Empowerment for people living with dementia: an integrative literature review. Int J Nurs Stud. 2021;124:104098. 10.1016/j.ijnurstu.2021.104098. 34706313 10.1016/j.ijnurstu.2021.104098

[52] Vermeer Y, Higgs P, Charlesworth G. What do we require from surveillance technology? A review of the needs of people with dementia and informal caregivers. J Rehabil Assist Technol Eng. 2019;6:1–12. 10.1177/2055668319869517. 10.1177/2055668319869517 PMC6891003 31832230

[53] Wangmo T, Lipps M, Kressig RW, Ienca M. Ethical concerns with the use of intelligent assistive technology: findings from a qualitative study with professional stakeholders. BMC Med Ethics. 2019;20(1):98. 10.1186/s12910-019-0437-z. 31856798 10.1186/s12910-019-0437-z PMC6924051

[54] Wöhlke S, Riedel A. Pflegeethik und der Auftrag der Pflege – Gegenwärtige Grenzen am Beispiel der stationären Altenpflege. Bundesgesundheitsblatt Gesundheitsforschung Gesundheitsschutz. 2023;66(5):508–14. 10.1007/s00103-023-03696-2. 37085589 10.1007/s00103-023-03696-2 PMC10121069

[55] Xu YA, Wang Y, Kim SSY, Kim DOD, Sun Y, McLaughlin ML. Safe at home: acceptance of surveillance technology among caregivers for persons with dementia. Health Informatics J. 2023;29(1):14604582231152188. 10.1177/14604582231152188. 36680337 10.1177/14604582231152188

[56] Yoon HK, Kim GS. An empowerment program for family caregivers of people with dementia. Public Health Nursing (Boston, Mass.). 2020:37(2):222–233. 10.1111/phn.12690. 10.1111/phn.12690 31797446

