# Supplementary material for: Between empowerment, patronization, and surveillance. A semi-structured interview study with persons with dementia and family caregivers on the empowering opportunities and perils of intelligent assistive technologies
Source: BMC Med Ethics. 2025 Apr 5;26:44. doi: 10.1186/s12910-025-01203-7 (PMC11971744; doi:10.1186/s12910-025-01203-7)
Supplement: Supplementary file 1 — Supplementary Material 1. [file 12910_2025_1203_MOESM1_ESM.docx]

**Interview guide with vignettes for informal caregivers**

**A. Questions about the participants' everyday life and caring activities**

A1. What do you think makes for good *[for professional caregivers: professional]* care?

→ To what extent are there special requirements for caregivers of people with dementia?

A2. When you think about the daily life and care of your sick relative *[for professional caregivers: your patients]:* What activities play a particularly important role for your relative/patient?

A3. To what extent do you seek help with your care? → See *[question]:* Who helps you? [for family caregivers only]

A4. Are there any technical devices that are important for your relative's health and safety, e.g. a home emergency call system? [for professional caregivers only: Are there any assistive technologies that you use regularly when caring for someone with dementia?] What is your attitude towards these devices?

A5. What do you think? Does the increasing technologization of care make a difference for male and female caregivers?

→ If so, how, and why?

→ If not, why not?

A6. And for younger or older caregivers?

→ If so, which and why?

→ If not, why not?

**B. Case Vignette: GPS Bracelet**

Consider the following situation: Mrs. M, in her early 70s, lives alone in a large flat. There are shops, a busy road, a forest, and a river nearby. Some time ago, Mrs. M. was diagnosed with dementia. Her adult children want to prevent Mrs. M. from getting lost when she goes shopping or for a walk. They suggest the use of a tracking system. This system is called a "tracking bracelet" and can be worn on the wrist like a watch *[interviewer points to picture 1].* The bracelet transmits so-called GPS signals, which can be used to locate it *[interviewer points to picture 1-2 .* When someone is wearing this bracelet, the wearer's location can be determined, as shown in the third picture. So if you ever get lost, a bracelet like this can help you to be found. *[The interviewer points to the appropriate places on the screen in picture 3).*

B1. What do you think of the use of this bracelet?

a) How does it affect Mrs. M's independence or autonomy?

b) How does this use affect Mrs. M's safety?

c) How does this intervention affect Mrs. M's privacy?

d) How does this intervention affect Mrs. M's quality of life?

e) How does this intervention affect Mrs. M's relationship with her children/friends/acquaintances?

f) What other effects might be possible?

B2. To what extent would it make a difference to your assessment if the bracelet were to alert a third party?

It is also possible that the wristband first learns Mrs. M's usual routes and then recognizes when she gets lost. It only sends information about where Mrs. M is when she strays from her usual routes. How would you rate a bracelet with this kind of functionality?

**C. Case Vignette: Emotion Recognition Technology**

Mr. F has advanced dementia. This has changed his personality. Sometimes he is in a bad mood or irritable. This sometimes leads to arguments or disputes with caregivers and nursing staff.

Research is now also being done on systems that can help in such situations,

e.g. technical emotion recognition systems. *[Interviewer describes the system and shows the comic/picture]:* The system you see here can detect early signs of bad mood or irritability through image and sound recordings. If the system detects such a situation *[interviewer points to picture 2],* the next step is to inform a caregiver *[interviewer points to picture 3].* The video or audio recordings are not transmitted. Appropriate solutions are offered *[interviewer points to the screen in the third picture] .*

C1. If you think about the points we have raised in the last few cases: How do you evaluate the use of such a system?

a. How does this involvement affect Mr. F's independence or autonomy?

b. How does this use affect Mr. F's safety?

c. How does this placement affect Mr. F's privacy?

d. How does this placement affect Mr. F's quality of life?

e. How does this intervention affect Mr. F's relationship with his caregivers?

f. What other effects do you think are possible?

C2. How do you assess the use of such a system in relation to the caregivers? How does it affect their privacy, sense of security, domesticity/working atmosphere?

a. How does this use affect the privacy of caregivers?

b. How does it affect the caregiver's sense of security?

c. How does it affect the working atmosphere?

d. What other effects can you think of?

C3. To what extent does it make a difference to you whether such a system is used in the home or in a nursing home?

C4. [For family caregivers]: Could you imagine using such a system for your relative?

→ If yes, why?

→ If not, why not?

What would it take for you to use such a system for your relative?

**D. Case vignette: Assistance with Dressing**

Mr. M. has advanced dementia. This makes it difficult for him, for example, to choose the right clothes and to remember the order in which to dress. There are now technical systems that can help.

**Option one: Smart Clothes Hangers**

For example, there are clothes hangers that help with dressing *[interviewer shows the comic strip].* These hangers have numbers on them *[interviewer points to pictures 2 and 3]* that indicate the order in which the clothes should be put on. For example, first the shirt *[picture 2]*  and then the jacket and tie *[picture 3] .* This ensures that the correct order is followed when, for example, going to the theatre *[picture 4].*

D1. When you think about the points we mentioned in the first case: How do you rate the use of this hanger?

a. How does it affect Mr. M's independence or autonomy?

b. How does this use affect Mr. M's safety?

c. How does this placement affect Mr. M's privacy?

d. How does this placement affect Mr. M's quality of life?

e. How does this intervention affect Mr. M's relationship with his caregivers?

f. What other effects do you think might be possible?

D2. *[For family caregivers]:* Could you imagine your relative using a hanger like this?

→ If yes, why?

→ If not, why not?

→ What would your relative need to be able to use such a hanger?

**Option two: DRESS System**

There are now systems that can do more than just display the order. They can also help you choose clothes for a particular occasion. *[Interviewer shows the comic.]* With this system, you choose between different occasions on the robot. *[Interviewer points to Figure 1.].* The robot will then show you suitable clothes on a screen *[interviewer points to picture 3]* and recognize whether you have thought of everything. If you have forgotten an item of clothing, it can draw your attention to it. You can then, for example, go to the theatre dressed appropriately *[picture 4] .*

1. Thinking about the points raised in the last few cases: How do you rate the use of this robot?

a) How does this use affect Mr. M's independence or autonomy? b) How does this use affect Mr. M's safety?

c) How does this use affect Mr. M's privacy?

d) How does this use affect Mr. M's quality of life?

e) How does this intervention affect Mr. M's relationship with his caregivers?

f) What other effects do you think might be possible?

2. Would advise from such a robot be comparable to advice from caregiver?

→ If so, why?

→ If not, why not?

→ What would be needed for the advice to be comparable?

3. To what extent would it make a difference to your assessment if a professional care service or a caregiver could control the robot?

4. [for family caregivers]: Could you imagine your relative using such a robot?

→ If so, why?

→ If not, why not?

→ What would be necessary for your relative to use such a robot?

**Conclusion:**

Is there anything you would like to add or that we have not yet covered that you think is very important to what we have discussed so far?
