# Supplementary material for: Between empowerment, patronization, and surveillance. A semi-structured interview study with persons with dementia and family caregivers on the empowering opportunities and perils of intelligent assistive technologies
Source: BMC Med Ethics. 2025 Apr 5;26:44. doi: 10.1186/s12910-025-01203-7 (PMC11971744; doi:10.1186/s12910-025-01203-7)
Supplement: Supplementary file 2 — Supplementary Material 2. [file 12910_2025_1203_MOESM2_ESM.docx]

**Interview guideline for persons with dementia**

**A. Questions about the daily life and living situation of the respondents.**

A1. Think about your everyday life: What daily activities are particularly important to you?

A2. Do you have any hobbies/leisure activities that are important to you?

A3. To what extent do you use help in your daily life, e.g. for cleaning, cooking, chewing? *[Ask]:* Who helps you with these activities?

A4. Who are your main caregivers?

A5. How do you live now? *[Question]:* What feelings do you associate with being at home?

A6. Do you have any technical devices that are important for your health and safety, e.g. a home emergency call system? If so, how do you deal with it? If not, have you ever considered or rejected it?

A7. Have you ever had difficulty in finding your way in an unfamiliar environment? *[Ask again if yes]:* How did you find it?

**B. Case vignette: GPS Bracelet**

[If yes, for A7] You have just said that you used to have difficulties finding your way in unfamiliar surroundings.

Nowadays, there are technical devices that can locate you at any time. These devices can help you to find your way again when you are unsure or have lost your way.

For example, there is a device called a GPS bracelet. *[The interviewer shows the comic and explains how it works:* This system is called a "tracking bracelet" and can be worn on the wrist like a wristwatch *[interviewer points to picture 1)].* The bracelet sends out signals that can be used to locate the person *[interviewer points to picture 1-2].* When someone is wearing the bracelet, the location of the wearer can be determined, as shown in the third picture. So, if you get lost, a bracelet like this can help you to be found. *[The interviewer points to the appropriate locations on the screen in Figure 3.]*

*[If no for A7]:* You said earlier that you like to go for walks and that you have not had any problems with orientation so far. That pleases us. Many people who enjoy walking are afraid of getting lost as they get older. There are now technical devices that can locate you at any time. If necessary, the system can help you find the right way if you are unsure or lost.

For example, there is a GPS bracelet. *[The iInterviewer shows the comic and explains how it works:* This system is called a "tracking bracelet" and it can be worn on your wrist like a wristwatch *[interviewer points to picture 1].* The bracelet sends out signals that can be used to locate the person *[interviewer points to picture 1-2].*

If the wearer is wearing the bracelet, their location can be tracked, as shown in the third picture. So, if you ever get lost, a bracelet like this can help you to be found. *[Interviewer points to the appropriate locations on the screen in Figure 3.]*

B1. Could you imagine using such a bracelet yourself*? [If B1: Yes]:* B1a):

Would you wear the bracelet all the time?

*[If B1a):* No]: In what activities outside the home would you wear the bracelet?

B2. How would you rate the use of the bracelet *[if B1 is answered in the affirmative:* for yourself]?

a) How does this use affect your autonomy or independence *[if B1 is answered in the negative:* on the autonomy and independence of the sponsor]?

b) How does this use affect your safety *[if B1 is answered in the negative: the safety of the wearer]?*

c) How does this use affect your privacy *[if B1 is negative: the privacy of the wearer]?*

d) How does this use affect your quality of life [*if B1 is answered in the negative: the quality of life of the wearer*]?

e) How does this assignment affect your relationship with your caregivers *[if B1 is negative: the relationship between the wearer and his/her caregivers]?*

B3. Who should be able to see where you are with the help of the *bracelet [if B1 is answered in the negative: the wearer is where he/she is]? [Interviewer explains again the technical possibilities of localization if necessary].*

B4. There is also the possibility that the bracelet first learns your usual routes and then recognizes when you get lost. It only sends information about where you are when you stray from your usual routes How would you rate a bracelet with this kind of functionality?

**C. Case Vignette: Emotion Recognition Technology**

C1 Sometimes people are in a bad mood or irritable. This can lead to arguments or disputes with people you spend a lot of time with. Do you know such situations?

*[If C1 no]:* That's good. If you could imagine such situations: What would you want in such a situation?

There are now technical systems that can help in such situations, e.g. technical emotion recognition. *[Interviewer describes the system and shows the co-mic/picture]:* The system you see here can detect early signs of bad mood or irritability through image and sound recordings. If the system detects such a situation *[interviewer points to picture 2],* the next step is to inform a caregiver *[interviewer points to picture 3].* The image or sound is not transmitted. Only appropriate solutions will be offered *[interviewer points to the screen in picture 3].*

*[If C1 yes]:*

a) Can you tell us how you feel in such situations?

b) How do you deal with such situations?

c) Are there any solutions that you have developed - perhaps together?

There are now technical systems that can help in such situations, e.g. technical emotion recognition. *[Interviewer describes the system and shows the comic/picture]:* The system you see here can recognize early signs of bad mood or irritability by means of image and sound recordings. If the system detects such a situation *[interviewer points to picture 2],* the next step is to inform a caregiver *[interviewer points to picture 3].* The image or sound is not transmitted. Only appropriate solutions will be offered *[interviewer points to the screen in the third picture].*

C2. Could you imagine using such a system in your home?

C3. How would you assess the use of the wristband *[if you answered C2 in the affirmative: for yourself]?*

a) What would be the impact of this assignment on your autonomy or independence [*if C2 is negative: on the autonomy and independence of the wearer]?*

b) How would this use affect your safety *[if C2 is denied: the safety of the wearer]?*

c) How would this use affect your privacy *[if C2 is denied: the privacy of the wearer]?*

d) How would this use affect your quality of life *[if the answer to C2 is no: the quality of life of the wearer]?*

e) How does this use affect the relationship with your caregiver *[if C2 is answered in the negative: the relationship between the wearer and the caregiver]?*

C4. How would your caregivers feel about using such a system?

**D. Case Vignette: Assistance with Dressing**

You said earlier that you like to do *[activity mentioned by the respondent].* Now there are people who sometimes find it difficult to choose the right clothes before leaving the house. For example, because their eyesight is getting worse with age, or because they can no longer concentrate as well. Has this ever happened to you?

No: That's good news. Imagine you were having difficulties. What would help you?

Yes: How did you manage back then? Do you have any strategies? Do you already have

Do you get support with your dressing?

...very interesting, it's great that it works/that you have someone to help you. There are now technical systems that can help.

**Option one: Smart clothes hangers**

For example, there are clothes hangers that help with dressing *[interviewer shows comic strip].* These hangers have numbers on them *[interviewer points to pictures 2 and 3]* that indicate the order in which the clothes should be put on. For example, first the shirt *(picture 2)* and then the jacket and tie *(picture 3).* This ensures that the correct order is followed when, for example, going to the theatre *(picture 4).*

D1 Could you imagine using such a hanger yourself?

D2 How would you rate the use of the dressing aid *[if D1 is answered in the affirmative: for yourself]?*

a) How does this use affect your autonomy or independence *[if D1 is answered in the negative: on the autonomy and independence of the user]? [Question]:* Would you feel patronized?

b) What effect does this use have on your self-confidence *[if D1 is negative: on the user's self-confidence]?*

c) How does this use affect your safety *[if D1 is answered in the negative: the safety of the user]?*

d) How does this use affect your privacy *[if D1 is answered in the negative: the user's privacy]?*

e) How does this use affect your quality of life *[if D1 is negative: the user's quality of life]?*

f) How does this use affect your relationship with your caregivers [*if D1 is negative: the user's relationship with his/her caregivers]?*

**Option two: DRESS system**

Systems are now being researched that can do more than just display the order. They can also help you choose clothes for a particular occasion. [*Interviewer shows the comic*] With this system, you choose between different occasions on the robot*. [Interviewer points to Figure 1].* The robot will then show you suitable outfits on a screen [interviewer points to picture 3] and check that you have thought of everything. If you have forgotten an item of clothing, it can draw your attention to it. You can then, for example, go to the theatre dressed appropriately [picture 4].

D' 1 Could you imagine using such a robot yourself?

D' 2 How would you rate the use of such a robot *[if D'1 is answered in the affirmative: for yourself]?*

a) How does this use affect your autonomy or independence *[if D'1 is answered in the negative: on the autonomy and independence of the user]?*

b) How does this use affect your self-confidence *[if D'1 is answered in the negative: on the self-confidence of the user]?*

c) How does this use affect your privacy *[if the answer to D'1 is no: the user's privacy]?*

d) How does this use affect your quality of life [*if the answer to D'1 is no: the user's quality of life]?*

e) How does this use affect your relationship with your caregivers *[if D'1 is answered in the negative: the user's relationship with his/her caregivers]?*

D'3 Would you use such a robot if a professional care service or your caregivers could control it? That is, choose the clothes for you?

D'4 Would the advice given by such a robot be comparable to the advice given by a caregiver or your relative?
