# Supplementary material for: Between empowerment, patronization, and surveillance. A semi-structured interview study with persons with dementia and family caregivers on the empowering opportunities and perils of intelligent assistive technologies
Source: BMC Med Ethics. 2025 Apr 5;26:44. doi: 10.1186/s12910-025-01203-7 (PMC11971744; doi:10.1186/s12910-025-01203-7)
Supplement: Supplementary file 3 — Supplementary Material 3. [file 12910_2025_1203_MOESM3_ESM.pdf]

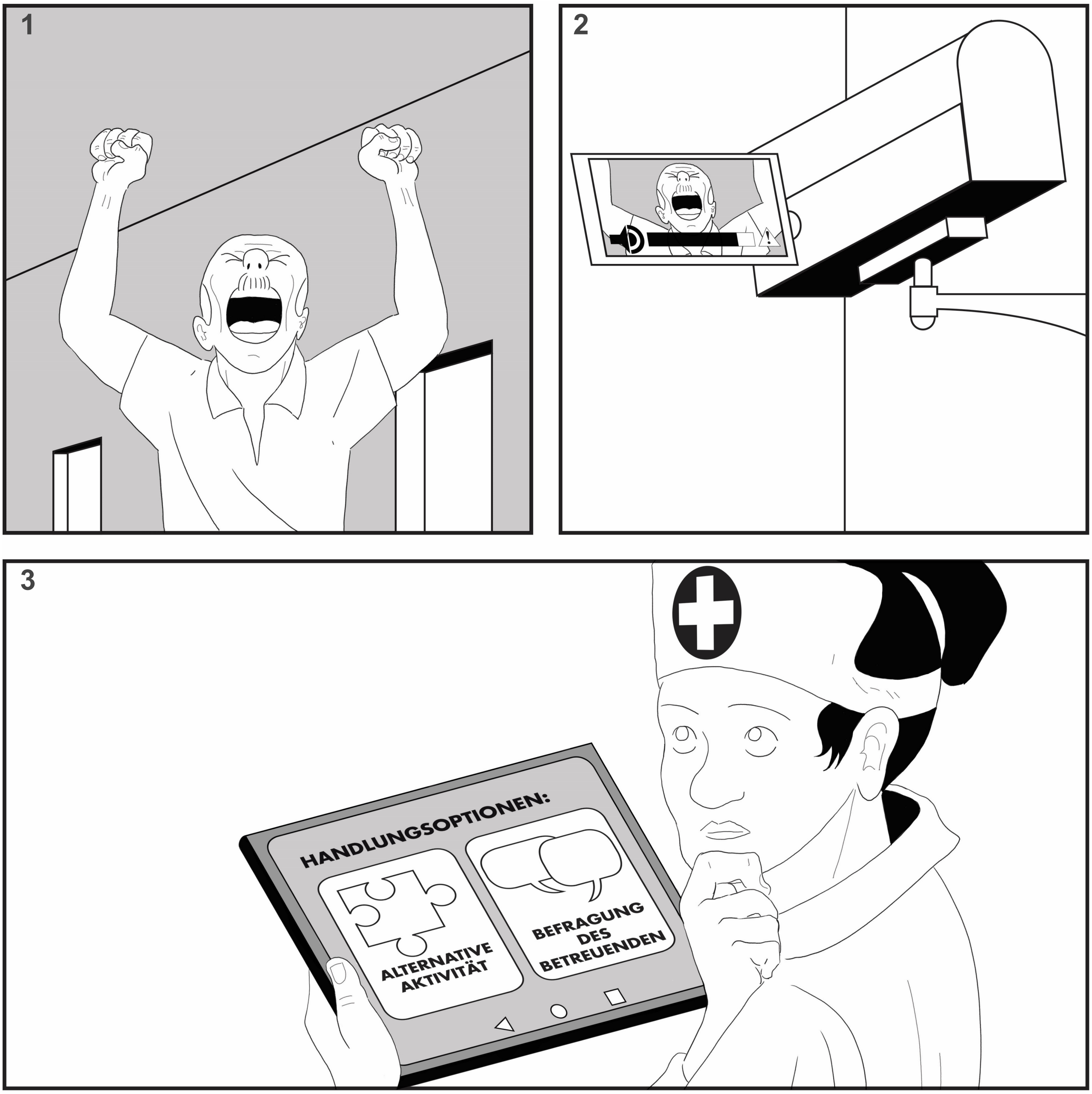

Figure S1 **Case Vignette B: Emotion Recognition Technology**

3 OPTIONS FOR ACTION:
A: ALTERNATIVE ACTIVITY
B: ASK CAREGIVER

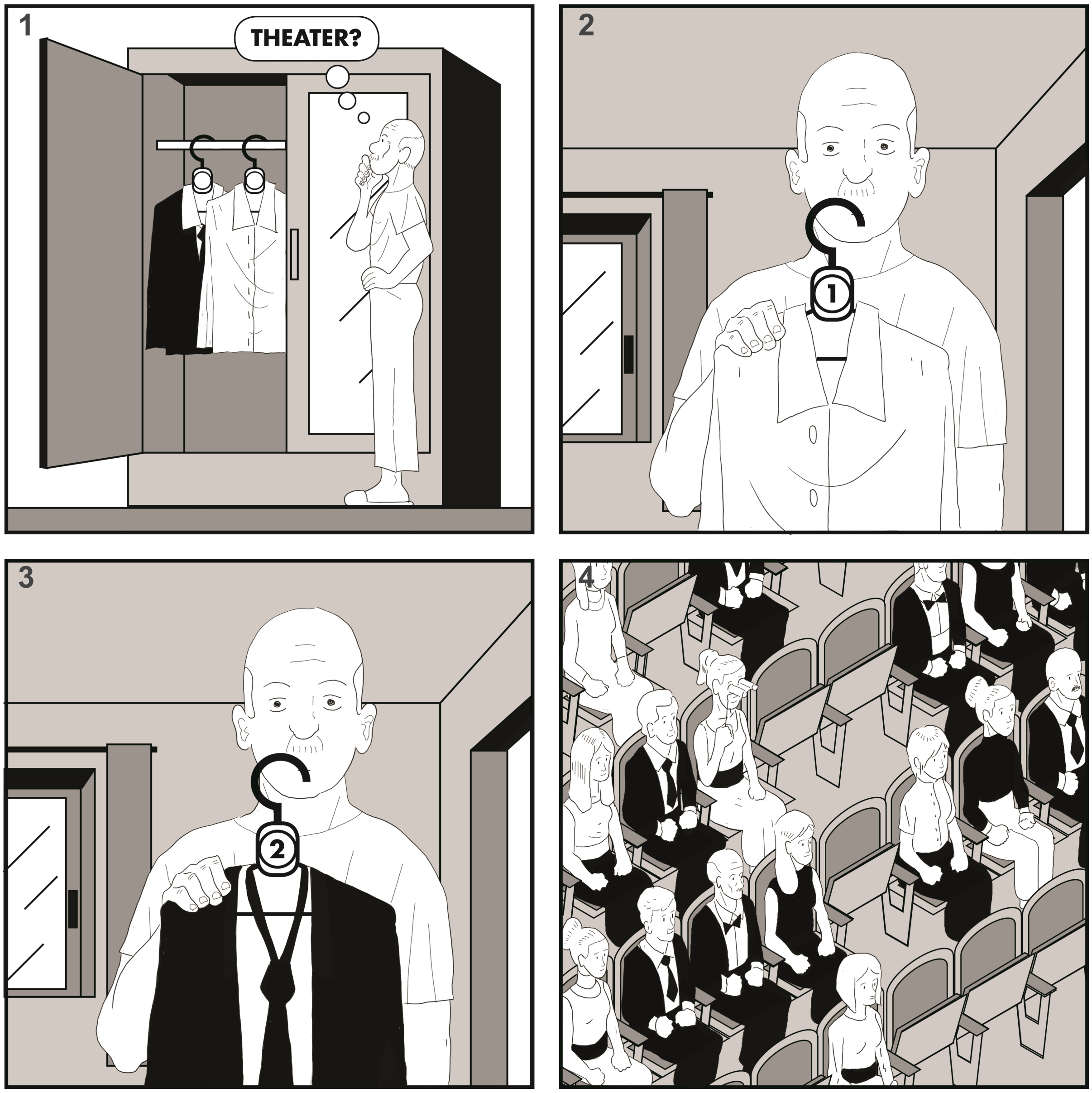

Figure S2 **Case Vignette C: Dressing Technology** *(Version 1: Smart Clothes Hangers)*

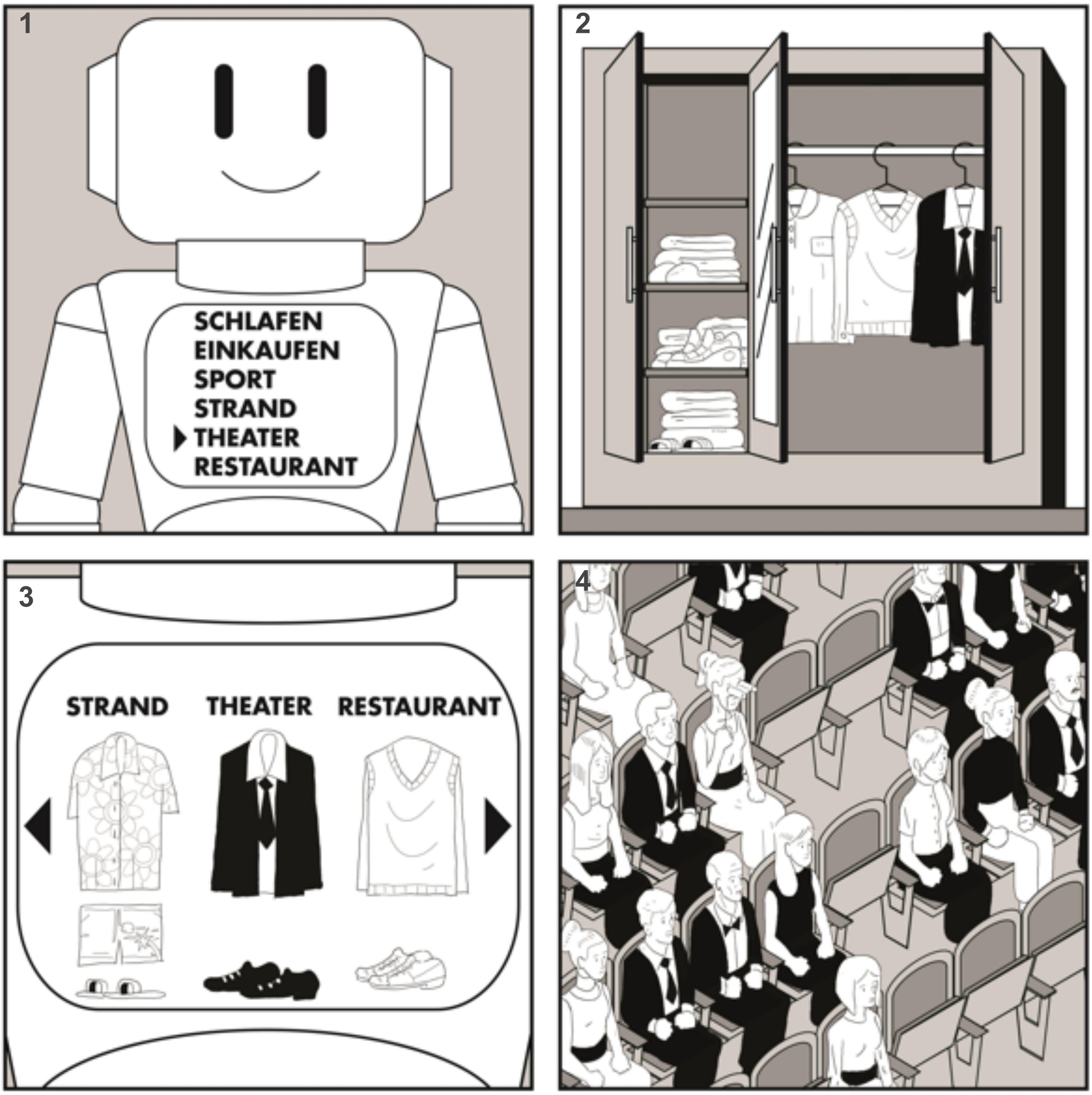

Figure S3 **Case Vignette C: Dressing Technology** *(Version 2: DRESS System)*

1 SLEEP
SHOPPING
SPORTS
BEACH
THEATER
RESTAURANT

3 BEACH
THEATER
RESTAURANT
